# Sex-Dependent QRS Guidelines for Cardiac Resynchronization Therapy Using Computer Model Predictions

**DOI:** 10.1016/j.bpj.2019.08.025

**Published:** 2019-08-28

**Authors:** Angela W.C. Lee, Declan P. O'Regan, Justin Gould, Baldeep Sidhu, Benjamin Sieniewicz, Gernot Plank, David R. Warriner, Pablo Lamata, Christopher A. Rinaldi, Steven A. Niederer

**Affiliations:** 1 School of Biomedical Engineering and Imaging Sciences, King’s College London, London, UK; 2 MRC London Institute of Medical Sciences, Imperial College London, London, UK; 3 Department of Biophysics, Medical University of Graz, Graz, Austria; 4 Department of Cardiology, Sheffield Teaching Hospitals NHS Foundation Trust, Sheffield, UK

## Abstract

Cardiac resynchronization therapy (CRT) is an important treatment for heart failure. Low female enrollment in clinical trials means that current CRT guidelines may be biased toward males. However, females have higher response rates at lower QRS duration (QRSd) thresholds. Sex differences in the left ventricle (LV) size could provide an explanation for the improved female response at lower QRSd. We aimed to test if sex differences in CRT response at lower QRSd thresholds are explained by differences in LV size and hence predict sex-specific guidelines for CRT. We investigated the effect that LV size sex difference has on QRSd between male and females in 1093 healthy individuals and 50 CRT patients using electrophysiological computer models of the heart. Simulations on the healthy mean shape models show that LV size sex difference can account for 50–100% of the sex difference in baseline QRSd in healthy individuals. In the CRT patient cohort, model simulations predicted female-specific guidelines for CRT, which were 9–13 ms lower than current guidelines. Sex differences in the LV size are able to account for a significant proportion of the sex difference in QRSd and provide a mechanistic explanation for the sex difference in CRT response. Simulations accounting for the smaller LV size in female CRT patients predict 9–13 ms lower QRSd thresholds for female CRT guidelines.

## Significance

Left ventricle size sex difference was identified as a mechanistic cause for cardiac resynchronization therapy (CRT) response sex differences. Computer models taking into account left ventricle size sex difference in CRT patients were used to predict female-specific QRS duration thresholds for current guidelines. Female-specific QRS duration thresholds ∼10 ms lower than current CRT guidelines, predicted using computer models, could potentially improve treatment options for female heart failure patients.

## Introduction

Cardiac resynchronization therapy (CRT) aims to improve cardiac function through resynchronizing electrical activation in heart failure patients. CRT is recommended (or considered) in patients with a reduced left ventricle (LV) ejection fraction (≤35%) and baseline electrical dyssynchrony measured using the QRS duration (QRSd) ≥130 ms (European Society of Cardiology (ESC) guidelines) or ≥150 ms (American College of Cardiology Foundation (ACCF)/American Heart Association (AHA) guidelines) with (or without) left bundle branch block (LBBB) (1, 2).

Clinical studies have found sex differences in CRT response at different QRSd. Males with wide QRSd (≥150 ms) had an improved CRT response compared to narrower QRSd (130–149 ms) values, whereas no such difference was reported in females (3). CRT response in males with wide QRSd (150–175 ms) was also found to be equivalent to female CRT response at lower QRSd (135–150 ms) (4).

Patients with narrow QRSd (<130 ms) are currently contraindicated for CRT treatment because these patients had increased mortality rates (5). However, there is growing evidence that females with narrow QRSd are at less risk of harm from CRT and may benefit from CRT treatment at shorter QRSd than males (3, 4).

Females in general had improved outcomes in response to CRT (3, 4); however, relatively fewer females receive CRT. Females have been underrepresented in CRT clinical trials, biasing the current CRT guidelines to males, which use the same QRSd thresholds for both sexes. This has led to interest in sex-dependent QRSd criteria for CRT. Although quantitative recommendations adapting the ACCF/AHA guidelines have been made (6), the specific mechanism to explain the sex difference in CRT response has not been demonstrated, with heart size, body size and height, conduction delays, or etiology of heart failure all being proposed as potential causes (7).

Varma et al. (4) proposed that the prolongation of the QRSd in LBBB patients was due to the decrease in the myocardial conductivity in the cardiac substrate and/or the increase in conduction path length. The conduction path lengthens with increasing LV size, which could change because of body size, sex, or heart failure remodeling, leading to LV dilation. Assuming that there is no sex difference in the underlying cause for reduction in the conduction in the cardiac substrate and, hence, response to CRT at the same level of cardiac substrate pathology, then the sex difference in response to CRT at different QRSd would arise from the LV size sex difference.

Females have been shown to have a smaller LV size and QRSd in healthy (8) and patient (4) populations. The correlation between the baseline healthy LV size and QRSd (8) and the sex difference in LV size in CRT patients (4) means that QRSd thresholds in the current CRT guidelines may be indicative of a higher level of ventricular conduction abnormality in females. This offers a mechanistic explanation for CRT response sex differences at different QRSd (3).

Our hypothesis is that LV size is a significant contributor to CRT response sex differences at different QRSd. We tested this hypothesis using computer models of the heart in healthy and patient cases to isolate the LV size effects on QRSd. Initially, we confirmed that males and females have different-sized hearts and that LV size correlates with predicted QRSd. We then quantified the degree to which LV size explains sex difference in QRSd. Finally, QRSd recommendations in the current guidelines for CRT, which were primarily determined from males, were calibrated using computer models to predict the female QRSd threshold for CRT treatment, taking into account female LV size.

## Methods

### Data set: healthy cases

Cardiac magnetic resonance (CMR) of 1093 healthy individuals with no known cardiovascular diseases were acquired using a balanced steady-state free precession sequence (voxel size: 1.25 × 1.25 × 2 mm). Images were collected with written informed consent at Hammersmith Hospital (London, UK) with study protocols approved by local ethics committee as part of UK Digital Heart Project (https://digital-heart.org/). A sex-specific cardiac atlas from these images was derived (9). Mean shape models of the LV were obtained by averaging the male (n = 493) and female (n = 600) segmentations (9).

### Data set: CRT patients

CMR of 50 CRT patients were acquired with electrocardiogram-gated free-breathing steady-state free precession sequence (median voxel size: 0.89 × 0.89 × 1 mm) as standard care, before CRT implantation. CRT inclusion criteria were as follows: New York Heart Association (NYHA) class II–IV, LV ejection fraction <35%, QRSd >130 ms, and optimal medical therapy. Data were collected with written informed consent at St Thomas’ Hospital (London, UK) or Sheffield Teaching Hospital (Sheffield, UK) with study protocols approved by local ethics committees. The patient population was primarily elderly (69.3 ± 11.2 years), males (78%) with LBBB (92%), and with 46% ischemic etiology (10). In the 50 patient cases, the LV was semiautomatically segmented from CMR images by two observers. An automatic method was used to construct the three-dimensional models, and an averaged consensus model was used (10) (https://doi.org/10.6084/m9.figshare.5853948).

### Electrophysiology simulations

QRSd sex differences in healthy individuals and CRT patients were explored by simulating the LV electrical activation on the healthy mean shape and CRT patient models with an eikonal model using the Cardiac Arrhythmia Research Package (11).

**Figure 1:**
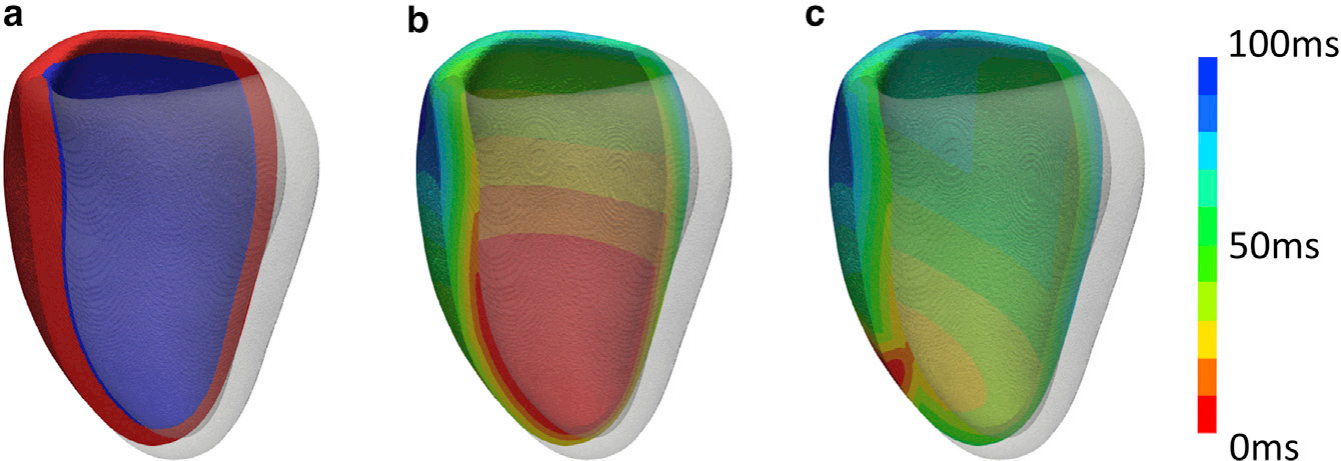
Mean shape model simulations. (*a*) Fast endocardial conduction model was used to simulate the electrical activity of the left ventricle. The endocardial layer (*blue*) had a sixfold increased CV compared to bulk myocardium (*red*). The electrical activation was then simulated for (*b*) intrinsic activation, in which the lower third of the endocardium was initially activated and (*c*) RV pacing was a surrogate for LBBB. To see this figure in color, go online.

A rule-based fast endocardial conduction model was used to simulate the electrical propagation of the left ventricle. Experimental studies have shown the conduction velocity (CV) in the myocardium varies across the ventricles with increased conduction in the endocardial layer (12). The heterogeneous cardiac substrate was modeled with a ∼1-mm thick layer on the endocardial surface, assumed to have sixfold increased CV in comparison to the bulk myocardium (Fig. 1 *a*). We have chosen to use eikonal equations that approximate the electrical activation of the heart (13). These equations allow for efficient simulations of activation patterns, allowing for larger numbers of simulations to be performed cost effectively.

The electrical activation of the ventricles was assumed to flow primarily along the myofibers, with the CV across the transverse fiber directions assigned to be 40% of the fiber direction CV. In normal human hearts, no sex differences in the gross fiber anatomy has been found (14), and fiber alignment has been shown to remain consistent in patients with hypertrophy or clinical heart failure (15). Although a sex difference in the fiber orientation could manifest in CRT patient populations, to date, we have no evidence to suggest that this happens. The LV fiber directions were therefore assumed to be consistent between male and female hearts and modeled using Bayer’s rule-based methods (16).

Ischemia was not accounted for in the models. Assuming that scarred regions have zero CV and the scar is not completely transmural, the electrical activation travels around the region of scar and thus has little effect on the QRSd. Although scarred regions have been found to have significant effects on the CRT response of patients, in this manuscript, our models are used to predict the QRSd sex difference rather than the CRT response of each patient. The QRSd sex differences under intrinsic and pathological electrical activation was investigated using mean shape models of healthy individuals and CRT patient models. To simulate non-LBBB, the ventricle was activated in the apical third of the endocardium (Fig. 1 *b*), where ex vivo studies have found a greater concentration of Purkinje fiber branching in healthy human hearts (17). LBBB was approximated by right ventricle (RV)-apex stimulation (Fig. 1 *c*) as studies have found there is no significant difference in the total LV activation time between RV-apex pacing and LBBB (18). In the model simulations, QRSd was approximated as the time taken for total LV activation time.

To predict the sex difference in the QRSd, we first estimated the CV in the male hearts that would result in a desired QRSd. This inferred CV was then used to predict how LV size sex difference affected the QRSd using female heart models in healthy and patient populations.

## Results

### LV size sex difference

The LV size for each model was quantified by LV mass and LV end diastolic volume (LVEDV). These LV size measures were determined for each mean shape model (male, female) and patient case (Table 1). LV mass was calculated by multiplying the volume of the mesh with the myocardial density (1.05 g/mL). We confirmed that the LV mass and LVEDV for healthy females was substantially smaller than males, consistent with literature (8). Our values for normal LV mass and LVEDV were reduced and increased outside of the range from literature values, respectively (8). This discrepancy likely is due to Lorenz et al. (8) including the papillary muscles in the LV mass, whereas we have included the papillary muscle in the LVEDV.

**Table 1:** LV Size Measures for the Mean Shape Models and the Patient Cases Differentiated into Male and Female Cohorts Compared Against Literature Values

Models or References	Patient Type	LV Mass (g)		LVEDV (mL)	
		Male	Female	Male	Female
Mean shape models	No HF^a^	138.9	114.7	158.1	124.9
Patient models (n = 50)	CRT	251.5	192.6	238.1	169.6
Lorenz et al. (8)	No HF	178 ± 31	125 ± 26	136 ± 30	96 ± 23
Varma et al. (4)	CRT	273 ± 78	173 ± 58	261 ± 109	165 ± 67

a No HF, No heart failure or known cardiovascular diseases.

We also confirmed the LV size sex difference was present in the CRT patient cohort, in which mean LV mass and LVEDV was statistically significantly smaller for females than for males (193 g and 170 mL vs 252 g and 238 mL, *p* < 0.05), consistent with literature (4).

### Effect of LV size on the QRSd

**Figure 2:**
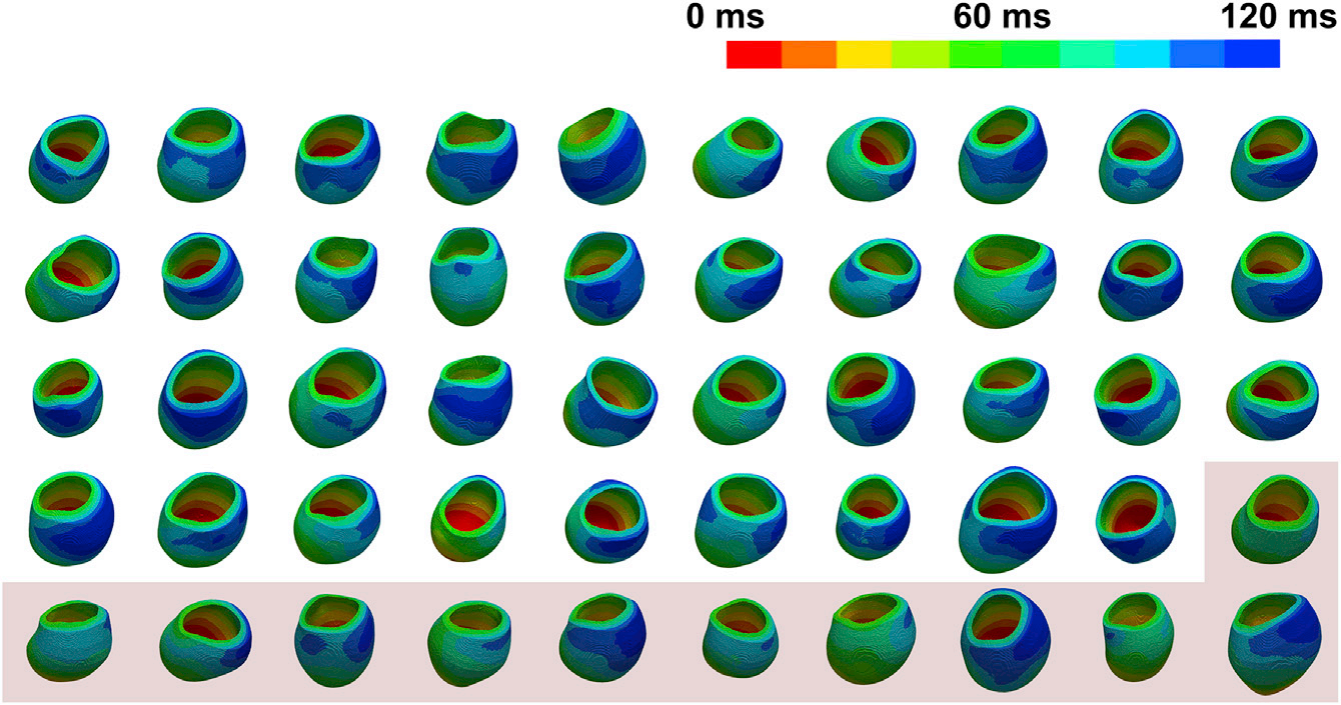
Patient model simulations. Intrinsic activation (non-LBBB approximation) was simulated for the male patient models (n = 39) to achieve a total activation time of 120 ms. The mean male CV was then applied to each of the female patient models (*red*, n = 11) to determine the equivalent female total activation times. To see this figure in color, go online.

**Figure 3:**
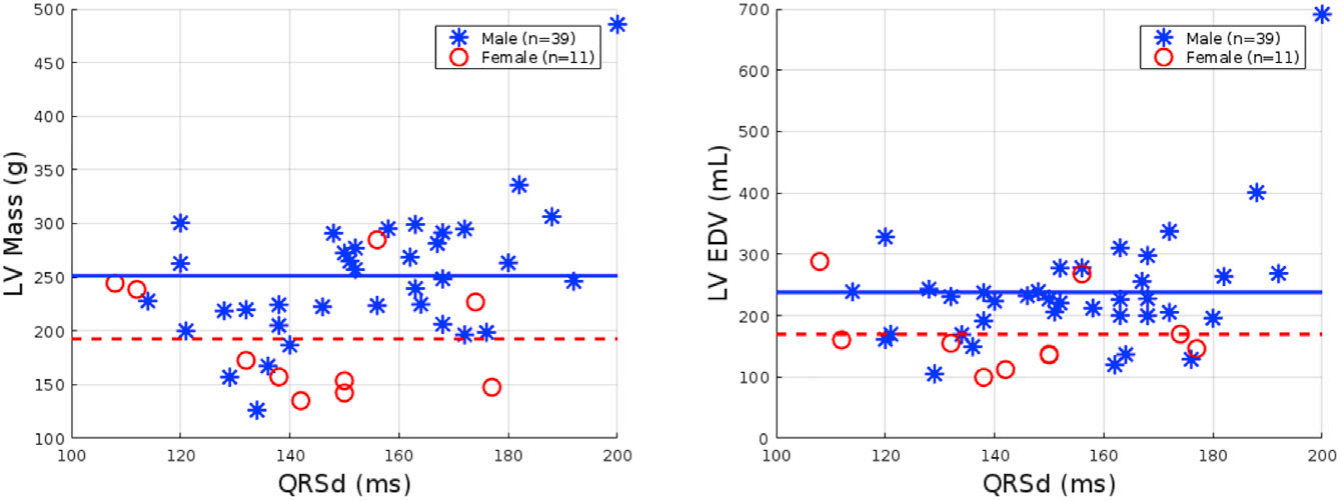
LV size measures in relation to the QRSd for the 50 patient cases. The mean LV size measures for the male (*blue stars*) and female (*red circles*) cohorts are shown as solid and dashed lines, respectively. To see this figure in color, go online.

Although LV size is a crucial factor in the QRSd, the location of the onset of electrical activation and the speed of the spread of electrical activation can also have an effect. Computer models allowed for the isolation and quantification of the effect of LV size on the QRSd across sexes for healthy and CRT patient cases (Fig. 2). In Fig. 3, the relationship between LV size and QRSd for each CRT patient case was plotted. The positive relationship between the QRSd and the LV size was confirmed in the CRT patient cohort, with Spearman correlation coefficients of LVEDV 0.21 and LV mass 0.36. Although these values are not consistent with literature values of 0.41 and 0.44, respectively (4), a similar trend of a positive correlation was observed, with the differences possibly due to the differences in enrollment criteria for the patient cohort, with the patient cohort used in this study having less strict enrollment criteria (NYHA class II–IV, ischemia allowed (46% of patients)) in comparison with Varma et al. (4) (NYHA III and nonischemic cardiomyopathy with true LBBB). The model predictions provide support for the collection of larger data sets of female patients with narrow QRSd. This would confirm the biophysical and statistical predictions (4) and provide more information to refine CRT patient selection.

### LV size and QRSd sex differences

Model simulations were used to identify if LV size sex difference could explain the QRSd sex difference observed in the healthy population. The CV for the mean male shape model needed to approximate an average male QRSd (90 ms (19) or 100 ms (20)) was applied to the mean female shape model. The LV size sex difference resulted in a female QRSd that was 4 ms lower, consistent with literature values of 4–8 ms reduction in QRSd values for females (90–86 ms (19) and 100–92 ms (20)).

### Calibration of QRSd for CRT patients on the basis of LV size sex difference

Female-specific QRSd thresholds for the CRT guidelines were estimated using the CRT patient models. LV electrical activation was simulated with RV-apex pacing (LBBB approximation) and intrinsic stimulation (non-LBBB approximation) for the male patients (n = 39). The CV required in each male patient to attain QRSd of 120, 130, and 150 ms was determined. The mean CV for males was then used in the female patients (n = 11) to predict the female-specific QRSd guidelines for LBBB and non-LBBB conditions (Table 2).

**Table 2:** Computer Models of the Male Patient Cases Were Used to Simulate the Electrical Activation of the LV with Intrinsic and RV Pacing

Male QRSd (ms)	Intrinsic Stimulation (Non-LBBB Approximation)		RV Pacing (LBBB Approximation)	
	Mean Male CV (m/s)	Female QRSd (ms) (n = 11)	Mean Male CV (m/s)	Female QRSd (ms) (n = 11)
120 ms	0.49	111.0 ± 10.8	0.65	109.1 ± 8.9
130 ms	0.45	120.2 ± 11.7	0.60	118.3 ± 9.7
150 ms	0.39	138.5 ± 13.5	0.52	136.8 ± 11.2

The CV needed to predict the male QRSd of 120, 130, and 150 ms were evaluated in each case. The mean male CV was applied to the female patient cases to predict the equivalent female QRSd values.

## Discussion

We used computational models to isolate the effect of LV size sex difference in QRSd in healthy individuals and CRT patients. Our simulations predicted that the LV size sex difference could explain 50–100% of the QRSd sex difference in healthy individuals. The simulations on the patient models predicted that current CRT guidelines should be reduced by ∼10 ms when applied to females.

### The role of LV size in QRSd sex difference

Literature studies on individuals without heart failure have shown that males have a larger LV and QRSd, with average healthy male QRSd 4–8 ms higher than females (19, 20). Our simulations with the mean shape models were consistent with literature, predicting a 4 ms shorter QRSd for females. This means our simulations predict that LV size sex difference can account for 50–100% of the QRSd sex difference.

In this study, we investigated the impact of size on QRSd. Other factors, including septal slowing, functional block, infarct size, and fast endocardial conduction ratio, could also impact QRSd. However, we have shown in a prospective clinical and simulation study (1) that these factors have a limited impact on the accuracy of simulating the electrical activation of the left ventricle and so were not considered in this study.

### Clinical relevance

CRT response sex differences have been found in clinical studies, with females having improved CRT response while being at less risk of harm at lower QRSd (3, 4, 6), leading to interest in sex-dependent QRSd guidelines for CRT. Consistent with our model predictions that the QRSd could be reduced to 137 ms for female patients with LBBB and mild heart failure, Zusterzeel et al. (6) proposed adapting the ACCF/AHA guidelines (1), reducing the QRSd threshold from ≥150 to ≥130 ms.

For moderate and severe forms of heart failure with LBBB, Zusterzeel et al. (6) proposed no sex difference, recommending lowering the QRSd (≥150 ms) for male and female patients to be in line with ESC guidelines (QRSd ≥130 ms). However, CRT response sex differences have also been found in moderate to severe cases of heart failure. Our models predict that the equivalent female QRSd thresholds for current guidelines were 9–13 ms lower than males for patients with non-LBBB and LBBB conditions with mild to severe heart failure (Table 3).

**Table 3:** Predicted Female QRSd Guidelines for CRT Recommendations Calibrated with LV Size

Guidelines	QRSd (ms)	QRS Morphology	EF^a^	ICM^b^	NYHA^c^ Class	COR^d^	Recommendation	Female QRSd (ms)
ESC 2016 (2)	>130	LBBB	<35%			I	Recommend CRT	>118
	>130	non-LBBB	<35%			II a	Consider CRT	>120
	<130		<35%			III	Contraindicated	<120
ACCF/AHA 2013 (1)	>150	LBBB	<35%		II, III, IV	I	Recommend CRT	>137
	>150	non-LBBB	<35%		II	II a	Can be useful	>138
	120–149	LBBB	<35%		II, III, IV	II a	Can be useful	109–136
	120–149	non-LBBB	<35%		III, IV	II b	May be considered	111–137
	>150	non-LBBB	<35%		II	II b	May be considered	>138
	>150	LBBB	<30%	ICM		II b	May be considered	>137
	<150	non-LBBB	<35%		I or II	III	Contraindicated	<138

a EF, Ejection fraction.

b ICM, Ischemic Cardiomyopathy.

c NYHA Class, New York Heart Association Functional Classification.

d COR, class of recommendation.

Our model simulations predict that CRT in females may be effective at QRSd <130 ms and that narrow QRSd (<130 ms) should be calibrated to <120 ms in females for the ESC guidelines (2). This is consistent with studies on CRT with narrow QRSd, in which unlike males, females with narrow QRSd did not have increased mortality rates with CRT (5). A recent study also found that a smaller LV size was associated with improved CRT response in patients with QRSd <130 ms (21), supporting lower narrow QRSd threshold for females who generally have smaller LV sizes.

Our models predict that a prolonged QRSd of 150 ms in males was equivalent to a QRSd of 137–138 ms in females. This was consistent with the Varma et al. study in which CRT response in males with wide QRSd range (150–175 ms) was equivalent to a shorter QRSd range (135–150 ms) for females (4). This was also consistent with studies that found distinct improvements in male CRT response as QRSd lengthens past 150 ms, whereas females have the same positive CRT responses with QRSd ≥ or <150 ms (3). Our models predict that a significant part of CRT response sex differences can be explained by smaller female heart sizes.

Our simulation results explain the sex difference in CRT treatment numbers (25% female) (22) as a subgroup of female patients who may benefit from CRT yet have QRSd ∼10 ms less than the current QRSd thresholds are presently not recommended for CRT. Incorporating female-specific QRSd thresholds into the guidelines could help clinicians in identifying females that could respond positively to CRT.

### Other factors influencing LV size

In this manuscript, we have shown that LV size sex difference affects the QRSd, a predictive measure for CRT response. LV size is influenced by factors other than sex, such as lifestyle choices, height, disease, race, and aging. Linde et al. recently concluded that in addition to QRSd, height also independently predicts CRT response, so height-corrected QRSd thresholds should be further explored (7). Smoking and diabetes have also been associated with an increase in LV mass. The impact of adult aging on the LV size is less clear, with studies having found either no change (19, 20) or an increase in the LV mass. There has also been debate into whether racial characteristics (African/Caucasian) effect the size of the LV with studies finding increased LV size for African subjects. Therefore, in addition to sex-dependent QRS guidelines for CRT, other patient subgroups may also benefit from distinct QRSd guidelines.

### Limitations

In this study, we have assumed that CRT response is dependent on a dyssynchronous electrical substrate. This assumption is based on the use of QRSd in current CRT inclusion criteria (1, 2) and that baseline QRSd and change in QRSd with pacing correlates with the response to CRT (3). However, QRSd is also a surrogate for activation pattern and material properties, which may be more predictive of CRT response. Assuming that this is the case, we have predicted new thresholds for CRT inclusion criteria, calibrating for differences in heart size between males and females. However, we have not tested these predictions, and the difference in CRT response between men and woman could be due to different as yet to be identified mechanisms.

We have assumed that the primary reason for the QRSd sex difference is because of the sex difference in the LV size and that the CV is consistent across genders. The CV is dependent on ionic channels in the cardiac cells. Although studies have found sex differences in cardiac ionic channels, these changes are primarily seen in repolarization channels of the heart (23) and do not affect the CV during the QRS complex.

However, CV in the cardiac substrate could also be affected by fibrosis, scar, and reduced intercellular coupling. Sex-dependent differences have been found in fibrosis, with fibrosis being less prevalent in female heart failure patients. It has been suggested that estrogen may be a modulating factor on the underlying pathways for fibrosis, which changes post-menopause as estrogen levels decline (24). In macroscopic modeling terms, fibrosis would result in reduced CV in the myocardium and hence a longer QRSd. This is represented in our models in Table 2. The CV was set to be constant between the sexes, and we are assuming that at the same level of fibrosis (or other cause of decrease in CV), the response to CRT would be the same regardless of gender.

On the other hand, if the underlying cause of response to CRT at prolonged QRSd is not dependent on the fibrosis, then the heightened male prevalence for fibrosis would only exacerbate the issue in which female patients would have a higher CV in comparison to male patients, leading to a further decrease in the female QRSd threshold. In addition to this, the sex difference in the prevalence of fibrosis in patients could also potentially be a cause for the lower enrollment number of female patients in CRT.

In this study, we investigated the impact of size on QRSd. Other factors, including septal slowing, functional block, infarct size, fast endocardial conduction, could also impact QRSd. We have shown in a prospective clinical and simulation study (13) that these factors have a limited impact on the accuracy of simulating the electrical activation of the left ventricle and so were not considered in this study.

In the models, it was assumed that male and female hearts followed the same fiber orientation rules as described by Bayer et al (longitudinal fiber orientation: +40° to −50° across the myocardial wall) (16) as in normal human hearts, gross fiber anatomy is equivalent between sexes (14), and no changes in fiber angle have been reported in hypotrophy or heart failure hearts (15). However, these measurements are made with a finite precision, and small differences in fiber angle could affect simulations results. We ran additional simulations to test the effect of perturbing the fiber angles by ±10% in the mean shape models of the male or female hearts. The CV was fitted to a QRSd of 100 ms for the male models. Fiber angle perturbations of ±10% was found to result in ±3 ms change in the QRSd in the female heart. Although this is only a ∼3% difference in the QRSd, this difference is in the same order of magnitude as the total difference in the mean QRSd between normal male and female hearts (4–8 ms). This makes it possible that subtle differences in fiber angle between male and female hearts may play a role in determining QRSd. To date, there is no evidence that indicates that a sex difference is present, but systemic or gender-specific differences in fiber orientation disease response may affect activation time and pattern.

In the model simulations, we have assumed a fixed anisotropy ratio of 0.4, in which the CV transverse to the fibers was 40% of the CV along the fibers (13). We tested this assumption in the healthy male and female models by perturbing the anisotropy ratio by ±10% and found that this resulted in only minor differences in the QRSd (<0.1 ms).

In contrast to previous studies using bidomain or monodomain simulations, an eikonal model was used to simulate the spread of the electrical activation in the LV to minimize the computational costs. In a previous study, the accuracy of the activation times for the eikonal model was within 1% of the reaction-eikonal model (13), which has been shown to be comparable to the bidomain models in simulating electrical activation of the heart (25).

## Conclusion

In this study, we used computer models to show that the sex difference in the QRSd can be explained with LV size sex difference. Computer models of CRT patients divided into male and female cohorts were used to identify the QRSd threshold values for the female patient population for CRT recommendations. Sex-dependent guidelines for CRT could help clinicians in better identifying female heart failure patients who could potentially benefit from CRT.

## Author Contributions

Conception and design or analysis and interpretation of data or both, A.W.C.L., D.P.O’.R., G.P., D.R.W., and P.L.; Drafting of the manuscript or revising it critically for important intellectual content, A.W.C.L., D.P.O’.R., B. Sidhu, B. Sieniewicz, G.P., D.R.W., P.L., C.A.R., and S.A.N.; Final approval of the manuscript submitted, C.A.R. and S.A.N.
